# Influence of marker-selection method in radiostereometric analysis of total knee arthroplasty on tibial baseplate migration patterns: a secondary analysis of a randomized controlled trial with 5-year follow-up

**DOI:** 10.2340/17453674.2024.40184

**Published:** 2024-03-21

**Authors:** Thies J N VAN DER LELIJ, Lennard A KOSTER, Perla J MARANG-VAN DE MHEEN, Sören TOKSVIG-LARSEN, Rob G H H NELISSEN, Bart L KAPTEIN

**Affiliations:** 1 Department of Orthopaedics, Leiden University Medical Center, Leiden, The Netherlands; 2 Safety & Security Science and Centre for Safety in Healthcare, Delft University of Technology, Delft, The Netherlands; 3 Department of Orthopaedics, Hässleholm Hospital, Hässleholm, Sweden; 4 Department of Clinical Sciences, Lund University, Lund, Sweden

## Abstract

**Background and purpose:**

Different marker-selection methods are applied to represent implant and tibial segments in radiostereometric analysis (RSA) studies of total knee arthroplasty (TKA). Either a consistent set of markers throughout subsequent RSA examinations (“consistent-marker method”) is used or all available markers at each follow-up (“all-marker method”). The aim of this secondary analysis was to compare marker-selection methods on individual and group level TKA migration results.

**Methods:**

Data from a randomized RSA study with 72 patients was included. Tibial baseplate migration was evaluated at 3 months, 1, 2, and 5 years postoperatively with both marker-selection methods. Additionally, migration was calculated using 5 fictive points, either plotted based on the consistent set of markers or all available markers.

**Results:**

Migration could be calculated with both marker-selection methods for 248 examinations. The same prosthesis and bone markers (n = 136), different prosthesis markers (n = 71), different bone markers (n = 21), or different prosthesis and bone markers (n = 20) were used. The mean difference in maximum total point motion (MTPM) between all examinations was 0.02 mm, 95% confidence interval –0.26 to 0.31 mm. 5 implants were classified as continuously migrating with the consistent-marker method versus 6 implants (same 5 plus one additional implant) with the all-marker method. Using fictive points, fewer implants were classified as continuously migrating in both marker-selection methods. Differences between TKA groups in mean MTPM were comparable with both marker-selection methods, also when fictive points were used.

**Conclusion:**

Estimated group differences in mean MTPM were similar between marker-selection methods, but individual migration results differed. The latter has implications when classifying implants for estimated risk of future loosening.

Radiostereometric analysis (RSA) is a technique to detect early implant migration, which is predictive for future loosening of tibial components in total knee arthroplasty (TKA) [1,2].

Marker-based RSA requires the bone and prosthesis to be defined in 3 dimensions by inserting small radiopaque markers in each segment [3]. However, marker projections can be superimposed by implant projection, be out of view due to incorrect patient positioning, be invisible due to poor roentgen technique, and individual markers can be unstable. An RSA analyst can choose to calculate migration using only those markers consistently visible at all RSA examinations (“consistent-marker method”) or use all available markers at each follow-up examination that can be matched to the reference RSA image (“all-marker method) (Figure 1). Interestingly, most RSA studies do not specify the marker-selection method used.

**Figure 1:**
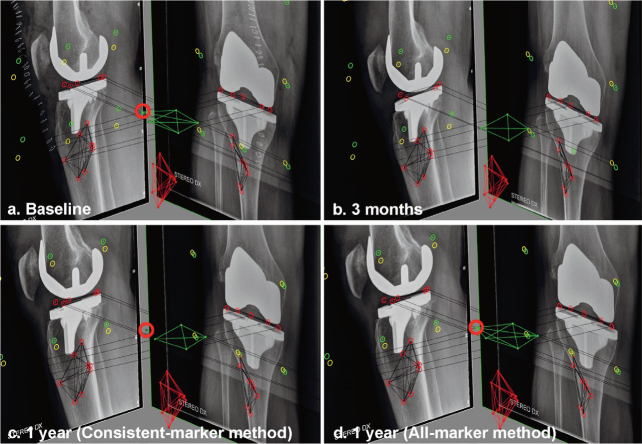
RSA examinations showing the baseline (a) and 3 months (b) follow-up examination of a patients. At 3 months, the anterolateral marker (red circle) in the PE insert is overprojected by the implant in the lateral radiograph. Using the consistent-marker method, only 4 of the 5 markers in the PE insert are selected at the 1-year follow-up (c) for the migration calculation (as these are the markers used at the 3 months examination). With the all-marker method, all 5 available markers that can be matched to the reference examination are used for the migration calculation at the 1-year follow-up (d).

For the maximum total point motion (MTPM), i.e., the length of the translation vector of the point in a rigid body that has the greatest motion, RSA guidelines and the ISO standard (ISO 16087:2013) state that if the points of measurement in a rigid body do not correspond between different implant designs, any comparison will become incorrect [4,5]. To overcome this problem, fictive points should be used to assess MTPM [4]. However, in RSA studies with markers in the polyethylene (PE) liner of the tibial component fictive points are often not used [6-11]. A recent review of all RSA studies on tibial component migration categorized “modular PE marker” and “fictive point” as separate marker-based RSA techniques [12]. Furthermore, RSA guidelines do not provide guidance on the number and location of fictive points that should be used, or how these points should be plotted using the actual tantalum insert markers [4].

The aim of our study was to assess whether the marker-selection method affects the calculated migration of individual implants as well as the mean estimated migration results at group level. As a secondary analysis, we assessed the influence of using fictive points.

## Methods

Patient enrollment and the 2- and 5-year outcomes have been described elsewhere [13,14]. In short, 72 patients were randomized to either a cementless Tritanium Triathlon Cruciate Retaining (CR) fixed bearing TKA or a cemented Triathlon CR fixed bearing TKA (both Stryker, Mahwah, NJ, USA). All patients were operated on by a single surgeon (STL). 8 spherical tantalum markers (ø 0.8 mm, RSA Biomedical, Umeå, Sweden) were inserted into the tibia, and 5 were implanted in the PE insert at standardized positions.

### Radiostereometric analysis

The first RSA examination was performed 2 days after surgery and served as the reference for the migration measurements. Subsequent examinations were performed at 3 months, 1, 2, and 5 years postoperatively. Supine RSA radiographs were taken with a biplanar calibration cage (Cage 10, RSA Biomedical, Umeå, Sweden) with a 90° angle between radiographs and analyzed using model-based RSA (MBRSA) (v.4.2, RSAcore, Leiden, Netherlands) [4]. The precision of the RSA setup was 0.1 mm for translations and 0.1° for rotations [13]. The migration of the tibial baseplate relative to the tibial bone was assessed and MTPM was used as the primary outcome measure [5].

The clinical studies presenting the 2- and 5-year results used the consistent-marker method for the migration calculations (without fictive points) [13,14]. For this study, migration was recalculated based on the same RSA scenes by the same researcher (TvdL) but using the all-marker method. The only difference between the methods was therefore the set of selected markers in either the PE liner or the tibial bone. Thresholds for ME (≤ 0.35 mm) and CN (≤ 120) were used for both methods [5]. Individual implants were considered continuously migrating if MTPM was ≥ 0.3 mm (i.e., ≥ 0.1 mm/year) between 2 and 5 years (1,16-19]. Implants with ≥ 0.2 mm micromotion at 2 years but micromotion of < 0.3 mm between 2 and 5 years were considered “stabilized.”

### Statistics

The limits of agreement between the 2 marker-selection methods, defined as the mean ± 1.96 x standard deviation (SD), should be within ± 0.5 mm of translation or ± 0.8° of rotation for the measures to be considered equivalent [20]. These thresholds were chosen as these are considered the smallest clinically relevant values [4,21,22]. There is no specific threshold described in the literature to be the smallest clinically relevant value of MTPM. However, individual implants showing ≥ 0.2 mm migration (MTPM) in year 2 are generally considered at risk of loosening [1,23,24]. Therefore, we considered 0.2 mm a clinically relevant threshold for MTPM.

The mean difference in MTPM between marker-selection methods was calculated separately for examinations in which different prosthesis marker, bone markers, or both were used. A linear mixed-effects model (LMM), which deals effectively with missing values and takes within-subject correlation into account, was used to compare the migration of TKA groups for both marker-selection methods [25]. The model consisted of a group variable, a time variable, and an interaction term between the time and group variable. Patients were included as a random factor by using a random-intercept term and the remaining variability was modelled with a heterogeneous autoregressive order 1 covariance structure. The likelihood ratio test was used to test for a difference in mean migration, comparing this model with a model including only the time variable. Given its non-normal distribution, MTPM was log-transformed, computed as log10(MTPM+1). Presented values were back-transformed to the original scale in millimeters. The differences in mean MTPM between the TKA groups at each follow-up moment were calculated for the consistent-marker and all-marker methods, using the delta method for approximating the standard error of the transformed results. Descriptive RSA data is presented to illustrate the directions of mean translations and rotations for both marker-selection methods. Additionally, we assessed whether the classification of individual TKAs as continuously migrating or stabilized differed between the marker-selection methods. Means were reported with 95% confidence interval (CI) or with range if this was indicative for the direction of the differences. A P value < 0.05 was considered statistically significant. Analyses were performed using SPSS (v.25, IBM Corp, Armonk, New York, USA) and R software (v.4.2.1, R Foundation for Statistical Computing, Vienna, Austria).

### Secondary RSA analyses using fictive points

Additional migration analyses were performed using 5 fictive points on the insert. 5 standardized points were chosen in the polyethylene insert (midpoint anteriorly, anterolateral, anteromedial, and 2 posterior points on the medial and lateral curves of the insert) and used to calculate the MTPM. The fictive points were plotted in all follow-up examinations based on either the migration of the consistent set of actual markers or all available markers. Note that the actual implant markers are still needed to plot these fictive points and need to adhere to the CN and ME thresholds.

### Ethics, registration, funding, and disclosures

The original RSA study was approved by the Regional Ethical Review Board in Lund (entry no. 2015/8) and registered at clinicaltrials.gov (NCT02578446). All patients gave their informed consent prior to enrollment. The original RSA study was funded by Stryker but Stryker had no part in the design, conduct, analysis, and interpretation stated in this paper. The authors declare no competing interests. Complete disclosure of interest forms according to ICMJE are available on the article page, doi: 10.2340/17453674.2024.40184

## Results

Of the 72 patients, 2 patients had missing baseline radiographs in the cemented group and could not be analyzed [14]. Because the insert of 1 patient in the cementless group was exchanged to treat an early postoperative infection, this patient was also excluded from the analysis. From 69 patients, 259 follow-up RSA examinations were performed during the 5-year follow-up. In 2 patients, 6 markers were implanted in the PE insert instead of 5. In 3 patients, 9 instead of 8 markers were placed in the tibial bone (Table 1).

**Table 1:** Characteristics of the marker-selection methods using the RSA examinations of a randomized controlled trial (RCT) including the 3 months, 1 year, 2 years, and 5 years follow-up examinations

Characteristics	Consistent-marker method	All-marker method
Patients, n	69	69
RSA examinations, n	248	250
Tibial prosthesis markers, n (%)		
3	87 (35)	34 (14)
4	94 (38)	93 (37)
5	67 (27)	119 (48)
6	0 (0)	4 (2)
ME prosthesis, mean (SD)	0.09 (0.05)	0.10 (0.05)
range	0.02–0.34	0.02–0.35
CN prosthesis, mean (SD)	37.8 (13.5)	32.0 (10.9)
range	21.5–102.9	21.2–102.9
Tibial bone markers, n (%)		
3	3 (1)	1 (0)
4	6 (2)	6 (2)
5	26 (11)	18 (7)
6	27 (11)	19 (8)
7	78 (32)	74 (30)
8	97 (39)	121 (48)
9	11 (4)	11 (4)
ME tibial bone, mean (SD)	0.14 (0.06)	0.15 (0.06)
range	0.03–0.33	0.03–0.33
CN tibial bone, mean (SD)	38.2 (11.6)	36.7 (10.9)
range	24.0–93.2	24.0–93.2

ME = mean error; CN = condition number.

In 9 RSA examinations the migration could not be calculated with either the consistent-marker or the all-marker method, because of inferior radiograph quality, or < 3 markers were visible. Tibial baseplate migration was calculated in 248 and 250 RSA examinations for the consistent-marker and all-marker method, respectively (Table 1). This difference was caused by 2 RSA examinations in which the specific set of same markers was not available to calculate migration, but sufficient other markers could be used to calculate migration with the all-marker method. Only in 29 (42%) of the 69 patients were the same prosthesis and bone markers used during all available follow-up examinations with both methods. For 248 RSA examinations, the exact same prosthesis and bone markers were used in 136 (55%) examinations, different prosthesis markers in 71 (29%), different bone markers in 21 (8%), and different markers in both prosthesis and bone markers in 20 (8%) examinations.

### MTPM, translations, and rotations

**Figure 2:**
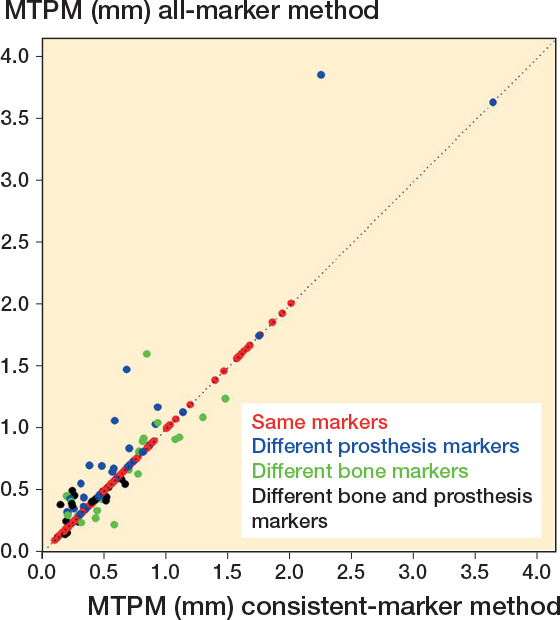
A scatterplot showing the MTPM of all RSA examinations calculated with both the consistent-marker and all-marker method (n = 248). The dotted line represents the line of equality. The difference in markers that have been used for the migration calculation for each examination is specified.

**Figure 3:**
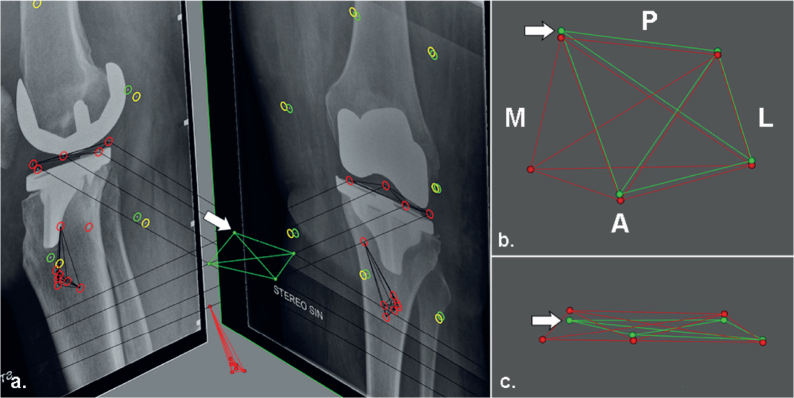
Figure showing the RSA examination with the greatest difference in MTPM between the consistent-versus all-marker method (a). The PE markers are shown from (b) superiorly and (c) anteriorly. Markers in red represent markers from the baseline image and green markers represent the markers from the specific follow-up examination. The difference in MTPM is cause by the medio-posterior marker (arrow), which is the marker that moved the most. In the consistent-marker method, this specific marker is not used for the migration calculation, because in another RSA examination of the same patients this marker was overprojected. As a result, the MTPM with the all-marker method is higher than with the consistent-marker method. A = anterior, P = posterior, M = medial, L = lateral.

The mean difference in MTPM of all examinations, including examinations in which the same prothesis and bone markers were used with both methods, was 0.02 mm (CI –0.26 to 0.31). The limits of agreements of MTPM exceeded the ± 0.2 mm thresholds (Table 2). When only different prosthesis markers were used, MTPM as calculated with the all-marker method was always equal or higher compared with the consistent-marker method (Figure 2). The “different prosthesis markers” group showed a mean difference in MTPM of 0.07 (range 0.00 to 1.61) between the marker-selection methods and included the examination with the greatest difference in MTPM between methods (Figure 3, see Appendix). In the group of examinations where only different bone markers were used, the mean difference in MTPM was 0.00 (range –0.35 to 0.76). Finally, in the subgroup of examinations where different markers in both prosthesis and bone were used, the mean difference was 0.04 (range –0.12 to 0.26). The limits of agreement for translations and rotations of all RSA examinations were within ± 0.5 mm and ± 0.8°, respectively (Table 2). The mean signed translations and rotations along and about each orthogonal axis showed comparable results with both marker-selection methods (Table 3, see Appendix).

**Table 2:** Differences in translations (mm), rotations (°), and MTPM (mm) between the consistent-marker and all-marker method and differences in migration results between the methods using 5 fictive points, matched either with a consistent set of actual markers or all available markers (secondary analysis). Mean differences are reported with the 95% confidence intervals (CI), which represent the limits of agreement between the 2 methods (n = 248 RSA examinations)

Method	Transverse	Translation (mm) Longitudinal	Sagittal	Transverse	Rotations (°) Longitudinal	Sagittal	MTPM
Differences between the consistent-marker and all-marker method							
mean (SD)	0.00 (0.09)	–0.01 (0.06)	0.01 (0.07)	0.02 (0.08)	0.00 (0.06)	0.00 (0.11)	0.02 (0.14)
CI	–0.17 to 0.17	–0.13 to 0.11	–0.14 to 0.15	–0.14 to 0.18	–0.13 to 0.12	–0.21 to 0.22	–0.26 to 0.31
Differences between the consistent-marker and all-marker method using 5 fictive points							
mean (SD)	0.00 (0.09)	0.00 (0.03)	0.01 (0.07)	0.02 (0.08)	0.00 (0.06)	0.00 (0.11)	0.01 (0.09)
CI	–0.17 to 0.16	–0.07 to 0.06	–0.13 to 0.15	–0.15 to 0.18	–0.12 to 0.12	–0.21 to 0.22	–0.27 to 0.29

**Table 3:** Mean translations (mm) along and rotations (°) about each orthogonal axis with 95% confidence intervals, as derived from the linear mixed-effects model

Axis Follow-up	Cemented (n = 34)		Cementless (n = 35)	
	Consistent-marker method	All-marker method	Consistent-marker method	All-marker method
Translation along transverse axis (mm)				
3 months	0.02 (–0.04 to 0.08)	0.01 (–0.06 to 0.07)	–0.05 (–0.11 to 0.00)	–0.05 (–0.12 to 0.01)
1 year	0.04 (–0.02 to 0.10)	0.06 (0.00 to 0.12)	–0.06 (–0.12 to 0.00)	–0.06 (–0.12 to 0.01)
2 years	0.06 (0.00 to 0.12)	0.05 (–0.02 to 0.11)	–0.07 (–0.12 to –0.01)	–0.06 (–0.12 to 0.01)
5 years	0.05 (–0.01 to 0.12)	0.01 (–0.06 to 0.08)	–0.06 (–0.12 to 0.00)	–0.05 (–0.12 to 0.01)
Translation along longitudinal axis (mm)				
3 months	0.02 (–0.04 to 0.08)	0.02 (–0.06 to 0.06)	–0.15 (–0.21 to –0.09)	–0.17 (–0.23 to –0.11)
1 year	0.02 (–0.04 to 0.08)	0.01 (–0.05 to 0.07)	–0.12 (–0.18 to –0.06)	–0.11 (–0.17 to –0.06)
2 years	0.01 (–0.05 to 0.07)	0.01 (–0.05 to 0.07)	–0.13 (–0.19 to –0.08)	–0.13 (–0.19 to –0.07)
5 years	0.03 (–0.03 to 0.10)	0.02 (–0.04 to 0.09)	–0.13 (–0.19 to –0.06)	–0.13 (–0.20 to –0.07)
Translation along sagittal axis (mm)				
3 months	0.02 (–0.07 to 0.12)	0.03 (–0.09 to 0.09)	0.02 (–0.08 to 0.11)	0.01 (–0.08 to 0.10)
1 year	0.06 (–0.04 to 0.15)	0.08 (–0.02 to 0.17)	0.00 (–0.10 to 0.09)	–0.01 (–0.10 to 0.09)
2 years	0.02 (–0.07 to 0.12)	0.03 (–0.07 to 0.13)	–0.03 (–0.12 to 0.07)	–0.01 (–0.11 to 0.08)
5 years	0.05 (–0.05 to 0.15)	0.06 (–0.04 to 0.16)	–0.01 (–0.11 to 0.08)	–0.03 (–0.12 to 0.07)
Rotation about transverse axis (°)				
3 months	0.01 (–0.23 to 0.26)	0.04 (–0.20 to 0.29)	–0.25 (–0.49 to –0.01)	–0.25 (–0.49 to –0.01)
1 year	–0.02 (–0.27 to 0.22)	0.01 (–0.23 to 0.26)	–0.40 (–0.64 to –0.16)	–0.39 (–0.62 to –0.15)
2 years	–0.11 (–0.35 to 0.15)	–0.08 (–0.33 to 0.17)	–0.44 (–0.68 to –0.20)	–0.44 (–0.68 to –0.19)
5 years	–0.10 (–0.36 to 0.16)	–0.07 (–0.33 to 0.19)	–0.42 (–0.67 to –0.17)	–0.42 (–0.67 to –0.17)
Rotation about longitudinal axis (°)				
3 months	–0.04 (–0.12 to 0.03)	–0.04 (–0.12 to 0.04)	0.01 (–0.07 to 0.08)	0.02 (–0.06 to 0.09)
1 year	–0.03 (–0.11 to 0.04)	–0.03 (–0.11 to 0.05)	–0.02 (–0.10 to 0.05)	–0.03 (–0.11 to 0.04)
2 years	–0.02 (–0.09 to 0.06)	–0.02 (–0.10 to 0.06)	–0.02 (–0.10 to 0.05)	–0.02 (–0.10 to 0.06)
5 years	–0.05 (–0.13 to 0.03)	–0.07 (–0.15 to 0.02)	0.03 (–0.05 to 0.11)	0.04 (–0.04 to 0.12)
Rotation about sagittal axis (°)				
3 months	0.02 (–0.08 to 0.12)	0.03 (–0.07 to 0.14)	0.13 (0.03 to 0.23)	0.12 (0.02 to 0.23)
1 year	–0.02 (–0.12 to 0.08)	–0.04 (–0.14 to 0.07)	0.11 (0.01 to 0.20)	0.11 (0.01 to 0.21)
2 years	–0.04 (–0.14 to 0.06)	–0.03 (–0.14 to 0.07)	0.11 (0.01 to 0.21)	0.12 (0.02 to 0.22)
5 years	–0.08 (–0.19 to 0.02)	–0.04 (–0.16 to 0.07)	0.04 (–0.06 to 0.15)	0.05 (–0.05 to 0.16)

### Group migration patterns

**Figure 4:**
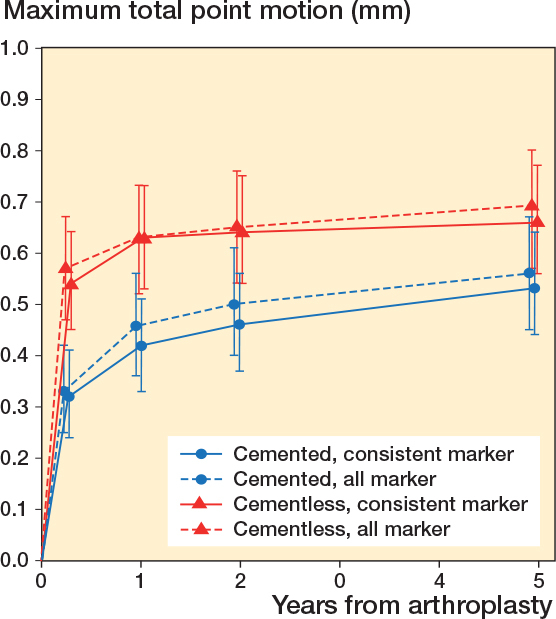
RSA results of maximum total point motion (MTPM). The mean and 95% confidence interval for the cemented and cementless group are shown for both the consistent-marker method and the all-marker method.

The estimated MTPM across all follow-up examinations, as derived from the LMMs, was slightly higher with the all-marker than with the consistent marker method (Table 4). Only at the 1-year follow-up for the cementless group, the mean MTPM was exactly the same. Regarding the estimated difference between TKA groups, the mean migration trajectory over the entire follow-up period of the cemented group was significantly higher compared with the cementless groups using either the consistent-marker method (P < 0.01) or the all-marker method (P < 0.01) (Figure 4). The difference in MTPM between TKA groups became smaller over time with both marker-selection methods.

**Table 4:** RSA migration analysis of maximum total point motion (MTPM) in the cementless and cemented group using either the consistent-marker or all-marker method and using 5 standardized fictive points, matched either with a consistent set of actual insert markers or all available markers at each follow-up moment. Values are mean MTPM (mm) with the 95% confidence intervals (CI), as derived from the linear mixed-effects model

Method Follow-up	Cementless	Cemented	Group difference
MTPM with consistent-marker method			
3 months	0.54 (0.45 to 0.64)	0.32 (0.24 to 0.41)	0.22 (0.09 to 0.35)
1 year	0.63 (0.53 to 0.73)	0.42 (0.33 to 0.51)	0.21 (0.07 to 0.34)
2 years	0.64 (0.54 to 0.75)	0.46 (0.37 to 0.56)	0.18 (0.04 to 0.32)
5 years	0.66 (0.56 to 0.78)	0.53 (0.43 to 0.64)	0.13 (–0.02 to 0.28)
MTPM with all-marker method			
3 months	0.57 (0.47 to 0.67)	0.33 (0.25 to 0.42)	0.23 (0.10 to 0.37)
1 year	0.63 (0.52 to 0.73)	0.46 (0.36 to 0.56)	0.17 (0.03 to 0.31)
2 years	0.65 (0.54 to 0.76)	0.50 (0.40 to 0.61)	0.14 (0.00 to 0.29)
5 years	0.69 (0.57 to 0.80)	0.56 (0.45 to 0.67)	0.13 (–0.03 to 0.29)
MTPM with fictive points plotted with consistent set of markers			
3 months	0.53 (0.44 to 0.64)	0.32 (0.23 to 0.41)	0.22 (0.08 to 0.35)
1 year	0.60 (0.49 to 0.71)	0.39 (0.30 to 0.48)	0.21 (0.07 to 0.35)
2 years	0.60 (0.49 to 0.71)	0.43 (0.33 to 0.53)	0.17 (0.03 to 0.31)
5 years	0.61 (0.51 to 0.73)	0.49 (0.39 to 0.60)	0.12 (–0.03 to 0.26)
MTPM with fictive points plotted with all available markers			
3 months	0.55 (0.44 to 0.65)	0.32 (0.23 to 0.41)	0.22 (0.09 to 0.36)
1 year	0.59 (0.49 to 0.71)	0.41 (0.31 to 0.51)	0.19 (0.04 to 0.33)
2 years	0.59 (0.49 to 0.71)	0.44 (0.35 to 0.55)	0.15 (0.00 to 0.30)
5 years	0.64 (0.53 to 0.75)	0.51 (0.40 to 0.63)	0.13 (–0.03 to 0.29)

### Continuously migrating implants

With the consistent-marker method, 5 implants (4 cemented and 1 cementless) were classified as continuously migrating (Table 5). Using the all-marker method, the same components and 1 additional cementless component were classified as continuously migrating. A larger increase in MTPM between 2 and 5 years with the all-marker method classified this additional implant as continuously migrating, because a different set of bone markers was used to calculate migration at 2-year follow-up, resulting in a lower MTPM. Both marker-selection methods classified 1 cementless implant as stabilized, but these were different implants (Table 5). Different bone markers were used in the migration calculation of patient 51, who was not classified as stabilized with the all-marker method as the migration in the second postoperative year was < 0.2 mm. Patient 7, where different prosthesis and bone markers were used, was not classified as stabilized with the consistent-marker method as the migration in the second postoperative year was < 0.2 mm and there were no 5-year follow-up results because the same set of markers was not available in those radiographs.

**Table 5:** Patients classified as stabilized or continuously migrating per group according to the marker-selection method

Factor	Cemented (n = 34)	Cementless (n = 35)
Stabilized ^a^		
Consistent-marker method	0	1 (# 51)
All-marker method	0	1 (# 07)
Fictive points		
Consistent marker set	0	1 (# 51)
All available markers	0	0
Continuously migrating ^b^		
Consistent-marker method	4 (# 09, 19, 40, 70)	1 (# 32)
All-marker method	4 (# 09, 19, 40, 70)	2 (# 16, 32)
Fictive points		
Consistent marker set	2 (# 40, 70)	0
All available markers	2 (# 40, 70)	1 (# 07)

a ≥ 0.2 mm MTPM in year 2 but < 0.3 mm between 2 and 5 years.

b ≥ 0.3 mm MTPM between 2 and 5 years.

### Secondary RSA analyses using fictive points

The mean differences in MTPM, translations, and rotations between the matching methods when using fictive points were comparable with the differences between the consistent-marker and all-marker method (Table 2). Fewer patients were classified as continuously migrating when fictive points were used (Table 5). Differences in MTPM between the TKA groups remained comparable (Table 4). Mean translations and rotations of the fictive point analyses are presented in Table 6 (see Appendix).

**Table 6:** Mean translations (mm) along and rotations (°) about each orthogonal axis with 95% confidence intervals, as derived from the linear mixed-effects model, when using 5 fictive points (FP) matched either with a consistent set of actual markers or all available markers in each follow-up

Axis Follow-up	Cemented (n = 34)		Cementless (n = 35)	
	Consistent-marker method	All-marker method	Consistent-marker method	All-marker method
Translation along transverse axis (mm)				
3 months	0.02 (–0.04 to 0.08)	0.01 (–0.06 to 0.07)	–0.05 (–0.11 to 0.00)	–0.05 (–0.12 to 0.01)
1 year	0.04 (–0.02 to 0.10)	0.06 (–0.01 to 0.12)	–0.06 (–0.12 to 0.00)	–0.06 (–0.12 to 0.01)
2 years	0.06 (0.00 to 0.12)	0.05 (–0.02 to 0.12)	–0.06 (–0.12 to –0.01)	–0.06 (–0.12 to 0.00)
5 years	0.05 (–0.01 to 0.11)	0.02 (–0.05 to 0.08)	–0.06 (–0.12 to 0.00)	–0.05 (–0.12 to 0.01)
Translation along longitudinal axis (mm)				
3 months	0.02 (–0.03 to 0.08)	0.02 (–0.04 to 0.07)	–0.17 (–0.22 to –0.11)	–0.18 (–0.23 to –0.12)
1 year	0.02 (–0.04 to 0.07)	0.01 (–0.04 to 0.07)	–0.14 (–0.20 to –0.09)	–0.14 (–0.20 to –0.09)
2 years	0.00 (–0.06 to 0.06)	0.00 (–0.06 to 0.05)	–0.15 (–0.20 to –0.09)	–0.15 (–0.20 to –0.09)
5 years	0.02 (–0.04 to 0.08)	0.02 (–0.04 to 0.08)	–0.13 (–0.19 to –0.07)	–0.14 (–0.20 to –0.09)
Translation along sagittal axis (mm)				
3 months	0.02 (–0.07 to 0.11)	0.03 (–0.06 to 0.12)	0.01 (–0.08 to 0.10)	0.00 (–0.09 to 0.10)
1 year	0.05 (–0.04 to 0.14)	0.08 (–0.02 to 0.17)	–0.01 (–0.10 to 0.08)	–0.01 (–0.10 to 0.09)
2 years	0.01 (–0.08 to 0.11)	0.03 (–0.07 to 0.12)	–0.02 (–0.12 to 0.07)	–0.01 (–0.10 to 0.09)
5 years	0.03 (–0.06 to 0.14)	0.05 (–0.05 to 0.15)	–0.02 (–0.11 to 0.08)	–0.02 (–0.13 to 0.07)
Rotation about transverse axis (°)				
3 months	0.02 (–0.23 to 0.23)	0.04 (–0.20 to 0.24)	–0.24 (–0.48 to –0.01)	–0.25 (–0.49 to –0.01)
1 year	–0.02 (–0.26 to 0.22)	0.01 (–0.23 to 0.25)	–0.40 (–0.64 to –0.17)	–0.39 (–0.63 to –0.15)
2 years	–0.11 (–0.35 to 0.14)	–0.08 (–0.33 to 0.16)	–0.44 (–0.68 to –0.20)	–0.44 (–0.68 to –0.19)
5 years	–0.09 (–0.35 to 0.16)	–0.07 (–0.03 to 0.19)	–0.42 (–0.66 to –0.17)	–0.42 (–0.66 to –0.17)
Rotation about longitudinal axis (°)				
3 months	–0.04 (–0.12 to 0.03)	–0.04 (–0.12 to 0.03)	0.01 (–0.06 to 0.09)	0.02 (–0.06 to 0.10)
1 year	–0.04 (–0.11 to 0.04)	–0.03 (–0.10 to 0.05)	–0.02 (–0.09 to 0.05)	–0.03 (–0.10 to 0.05)
2 years	–0.02 (–0.09 to 0.06)	–0.02 (–0.10 to 0.06)	–0.02 (–0.09 to 0.05)	–0.02 (–0.10 to 0.05)
5 years	–0.05 (–0.13 to 0.03)	–0.07 (–0.15 to 0.02)	0.02 (–0.05 to 0.10)	0.04 (–0.04 to 0.12)
Rotation about sagittal axis (°)				
3 months	0.02 (–0.08 to 0.12)	0.03 (–0.07 to 0.13)	0.13 (0.04 to 0.10)	0.12 (0.02 to 0.23)
1 year	–0.02 (–0.12 to 0.08)	–0.04 (–0.14 to 0.06)	0.11 (0.01 to 0.20)	0.11 (0.01 to 0.21)
2 years	–0.04 (–0.14 to 0.06)	–0.04 (–0.14 to 0.07)	0.11 (0.02 to 0.21)	0.12 (0.02 to 0.23)
5 years	–0.08 (–0.19 to 0.02)	–0.04 (–0.16 to 0.07)	0.04 (–0.06 to 0.14)	0.05 (–0.05 to 0.16)

## Discussion

We demonstrated that the mean difference in MTPM across all RSA examinations between the marker-selection methods was very small (0.02 mm, CI –0.26 to 0.31) and did not result in different conclusions regarding TKA group migration. However, the limits of agreement exceeded the clinically relevant threshold of ± 0.2 mm for individual implant migration. This implies that the two marker-selection methods cannot be used interchangeably when assessing individual migration patterns based on strict thresholds. We demonstrated that for most patients in at least one follow-up examinations a different marker-selection method resulted in different markers being used for the migration calculation.

The all-marker method resulted in equal or higher MTPM than the consistent-marker method when only prosthesis markers differed, which logically follows from the definition of MTPM. Using the all-marker method, the estimated MTPM can be determined by a marker that is not selected when using the consistent-marker method, but not vice versa. Therefore, MTPM with the consistent-marker method can never be higher than with the all-marker method if only different prosthesis markers are used. When different bone markers are used, MTPM can be either higher or lower with the all-marker method as this influences the alignment of the reference rigid body.

Mean MTPM is frequently used to assess the risk of loosening of TKA designs [2]. As we found the influence of marker-selection method on mean estimated MTPM to be small, it appears justified to compare group-level migration results across RSA studies using either method. However, the number of individual implants classified as continuously migrating according to specific thresholds is also frequently reported in RSA studies [19,23,26]. We showed that this number may depend on the marker-selection method that is used.

**Figure 5:**
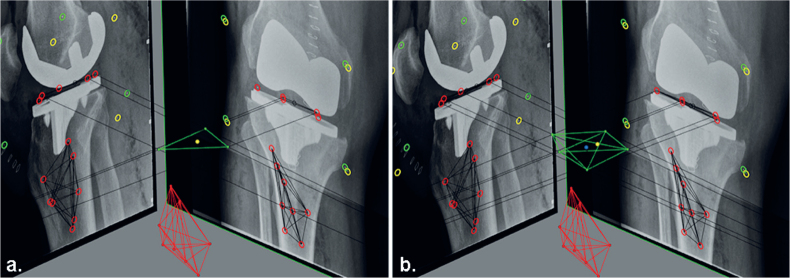
Figure showing the different set of makers of the migrating rigid body (prosthesis) in the reference RSA examination, as used for the migration calculations with (a) the consistent-marker method and (b) the all-marker method. The reference origin (geometrical center) for the migrating coordinate system that is used with the consistent-marker method (yellow point) differs from the reference origin that is used with the all-marker method (blue point).

In routine RSA, migration is expressed in a migrating coordinate system that has its origin in the geometric center of the prosthesis markers in the reference examination, and is aligned with the global coordinate system (Figure 5 see Appendix) [3,4]. Although MTPM and rotations are not affected by changing the origin of the migrating coordinate system, the latter does affect the calculated translations [3]. RSA guidelines state that the point(s) used to calculate translation of a rigid body should be “standardized” at all follow-up occasions in all patients [4]. However, this recommendation is ambiguous and does not provide clear guidance on which marker-selection method to use. Using the consistent-marker method, the origin of the implant will remain consistent between all follow-up examinations within the same patient. On the other hand, with the all-marker method, the origin differs between follow-up moments within the same patients. For both methods, the location of the origin will differ between patients, as the set of available markers differs.

Although RSA guidelines state that fictive points should be used to calculate MTPM, fictive points are not always used in clinical RSA studies. Nilsson et al. [27] described that by using the location of the actual insert markers on the radiographs, the positions of the plotted fictive points could be transformed to their corresponding position. However, it is unclear which fictive points should be used and which actual insert markers should be used to plot the position of the fictive points. The present study shows that using either a consistent set of markers or all available markers to plot the fictive points did not result in different conclusions regarding TKA group migration. However, it did influence the classification of individual implants.

More RSA examinations were used to calculate implant migration with the all-marker (n = 250) versus the consistent-marker (n = 248) method. A disadvantage of the all-marker method is that one individual migrating marker may influence MTPM. For example, a prosthesis marker in an RSA examination might be excluded in the next examinations when it leads to an ME threshold violation. In previous examinations this unstable marker may already have migrated relative to other markers in the same rigid body without exceeding the ME threshold. The MTPM of the previous examinations may then be erroneously high because of one unstable marker. The consistent-marker method would not have this problem, because the unstable marker will not be part of the set of consistent markers. A disadvantage of the consistent-marker method is that migration results of examinations can change because of subsequent RSA examinations, when the later follow-up examination is the limiting factor in determining the consistent set of markers. It may be counterintuitive that short-term migration results are affected by later examinations.

### Strengths

This was the first study to compare different marker-selection methods to calculate implant migration within a clinical RSA study. As one researcher (TvdL) performed all RSA analyses using the exact same RSA scenes for both marker-selection methods, there was no inter- or intra-observer variability that affected the calculated differences between the methods. Additionally, all patients were operated on by a single surgeon (STL) and there was no systematic difference in marker placement between patients. Moreover, the findings of our study are not restricted to marker-based RSA. Even model-based RSA still requires markers in the bone [30]. Our findings are also relevant for RSA of other joints, where different sets of markers at successive follow-up moments may also be present.

### Limitations

The quality of radiographs may differ between studies and it is possible that the proportion of examinations with different prosthesis and/or bone markers would be different in other studies. Note that if individual markers are excluded for specific reasons, such as overprojection or instability, results in structural differences could potentially change the resultant shape of the rigid body systematically. Finally, although it has its own limitations, CT-based RSA avoids the issue of marker-selection method [31].

### Conclusion

We showed that the estimated differences in migration at group level did not change when using either the all-marker or consistent-marker method, or when using 5 fictive points. However, individual implant migration measurements are different between marker-selection methods.

In perspective, RSA studies should report the marker-selection method that is used, as part of the standardized output to facilitate comparison between clinical studies. Moreover, if fictive points are used, the location of these points and how they were plotted needs to be reported.

## Notes

TvdL: data collection, RSA measurements, statistical analysis, interpretation of data, writing initial draft manuscript. BK, PM, LK: statistical analysis, interpretation of data. RN: interpretation of data. STL: study design and coordination of RCT, interpretation of data.

Handling co-editors: Keijo Mäkelä and Robin Christensen

*Acta* thanks Stuart Adam Callary and Stergios Lazarinis for help with peer review of this manuscript.
